# Rosiglitazone Alleviates Mechanical Allodynia of Rats with Bone Cancer Pain through the Activation of PPAR-*γ* to Inhibit the NF-*κ*B/NLRP3 Inflammatory Axis in Spinal Cord Neurons

**DOI:** 10.1155/2021/6086265

**Published:** 2021-08-25

**Authors:** Jie Fu, Baoxia Zhao, Chaobo Ni, Huadong Ni, Longsheng Xu, Qiuli He, Miao Xu, Chengfei Xu, Ge Luo, Jianjun Zhu, Jiachun Tao, Ming Yao

**Affiliations:** ^1^The Second Affiliated Hospital & Yuying Children's Hospital of Wenzhou Medical University/The Second School of Medicine, Wenzhou Medical University, Wenzhou, China; ^2^Department of Anesthesiology and Pain Research Center, The First Hospital of Jiaxing or The Affiliated Hospital of Jiaxing University, Jiaxing, China

## Abstract

Bone cancer pain (BCP) is a serious clinical problem that affects the quality of life of cancer patients. However, the current treatment methods for this condition are still unsatisfactory. This study investigated whether intrathecal injection of rosiglitazone modulates the noxious behaviors associated with BCP, and the possible mechanisms related to this effect were explored. We found that rosiglitazone treatment relieved bone cancer-induced mechanical hyperalgesia in a dose-dependent manner, promoted the expression of peroxisome proliferator-activated receptor-*γ* (PPAR-*γ*) in spinal cord neurons, and inhibited the activation of the nuclear factor-kappa B (NF-*κ*B)/nod-like receptor protein 3 (NLRP3) inflammatory axis induced by BCP. However, concurrent administration of the PPAR-*γ* antagonist GW9662 reversed these effects. The results show that rosiglitazone inhibits the NF-*κ*B/NLRP3 inflammation axis by activating PPAR-*γ* in spinal neurons, thereby alleviating BCP. Therefore, the PPAR-*γ*/NF-*κ*B/NLRP3 signaling pathway may be a potential target for the treatment of BCP in the future.

## 1. Introduction

Bone cancer pain (BCP) is one of the most common types of cancer-related pain that seriously affects the quality of life of cancer patients [1]. Approximately 90% of patients with advanced bone cancer must cope with chronic pain syndromes are associated with tumor progression and failed treatment [2]. Unfortunately, conventional pharmacologic therapies for BCP management are inadequate because of their limited analgesic effects or unavoidable side effects. Despite decades of basic and clinical research, the exact cellular and molecular mechanisms of cancer-induced pain remain elusive [3, 4], and there is a pressing need for more effective pharmacological treatments for this condition.

Rosiglitazone is a thiazolidinedione antidiabetic drug that acts as an insulin sensitizer by binding to peroxisome proliferator-activated receptor-*γ* (PPAR-*γ*) in adipocytes, making the cells more sensitive to insulin [5, 6]. Studies have shown that rosiglitazone plays a neuroprotective role in neurological disorders such as spinal cord injury, allergic encephalomyelitis, amyotrophic lateral sclerosis, Parkinson's disease, cerebral hemorrhage, and Alzheimer's disease [7–10]. In addition, rosiglitazone has promising therapeutic potential for the treatment of chronic pain, as it has been shown to ameliorate postincisional pain [11], neuropathic pain [12–14], and inflammatory pain [15, 16]. However, whether rosiglitazone relieves BCP remains unclear.

Nuclear factor-kappa B (NF-*κ*B) is a widely studied transcription factor that is involved in many physiological processes, such as regulating multiple aspects of innate and adaptive immune function [17, 18], and as a key mediator of the inflammatory response in regulating inflammatory pain [19], neuropathic pain [20], and other chronic pain [21]. Recent studies have shown that NF-*κ*B participates in the inflammatory cascade by activating the Nod-like receptor protein 3 (NLRP3) inflammasome in the nucleus [22, 23]. The activation of inflammasomes contributes to the development of chronic pain [24, 25]. This suggests that blocking the functional crosstalk between NF-*κ*B and NLRP3 may reduce chronic pain induced by bone cancer. Previous studies have shown that rosiglitazone may exert its anti-inflammatory and neuroprotective effects by inhibiting the NLRP3 inflammasome and reducing the expression of NF-*κ*B [26–28], but whether rosiglitazone can affect NF-*κ*B and NLRP3 in the spinal cord of rats with BCP to reduce pain needs further exploration.

This study investigated whether intrathecal injection of rosiglitazone reduced mechanical hyperalgesia induced by bone cancer and explored the underlying mechanism. We used a well-characterized BCP model that exhibited a stable and persistent pain state. We found that intrathecal injection of rosiglitazone reduced the mechanical hyperalgesia induced by bone cancer by activating PPAR-*γ* to inhibit the NF-*κ*B/NLRP3 inflammatory axis in spinal cord neurons. This will provide a new approach for the treatment of BCP.

## 2. Materials and Methods

### 2.1. Animals

Adult female Sprague-Dawley (SD) rats (aged six weeks, 160–180 g) were provided by Shanghai Legian Biotechnology Co., Ltd. All rats were housed at a constant room temperature (RT) of 23 ± 1°C on a 12 h light/dark cycle with free access to food and water. All animal experiments were conducted between 8:00 and 20:00. During the operation, a heating blanket was used to keep the body temperature stable, and all operators skillfully performed the related surgical procedures to shorten the operation time. With reference to previous studies [29, 30], sodium pentobarbital was prepared as a solution with a final concentration of 5 mg/ml to avoid irritation. After surgery, wounds were topically disinfected with 75% (*v*/*v*) ethanol, but not with systemic antibiotics, to avoid interference with experimental pharmacological treatments. As the purpose of the current study was to examine a chronic pain state, no analgesics were used during behavioral assessment to prevent distortion of the results. At the end of the experiment, the rats were euthanized using an excessive amount of anesthetics (sodium pentobarbital, 100 mg/kg) after being isolated from other rats. The researchers optimized the experimental design, selected the best experimental scheme, and reduced the number of animals whenever possible. All efforts were made to minimize animal suffering. All experimental procedures were approved by the Animal Use and Care Committee for Research and Education of Jiaxing University (Jiaxing, China) and the ethical guidelines for investigating experimental pain in conscious animals [31].

### 2.2. Preparation of Tumor Cells

The tumor cells were prepared according to a previously described protocol [32–34]. Walker 256 breast cancer cells were kindly provided by the Nanjing University of Chinese Medicine. Walker 256 breast cancer cells were intraperitoneally administered to female Sprague-Dawley rats and cancerous ascites developed after 6-7 days [35]. Carcinoma cells were extracted and immediately washed with a sterilized PBS solution. These cells were resuspended to a final concentration of 10^7^ cells/ml. Carcinoma cells of the same concentration that had been heat-killed for 30 min were used in the sham groups.

### 2.3. Establishment of a BCP Rat Model

The establishment of a BCP rat model was done as described in previous studies [36, 37]. Briefly, the rats were anesthetized via intraperitoneal injection with sodium pentobarbital (50 mg/kg). The superficial skin of the left hind limb was then scraped to expose the lower third of the rat's left tibia, disinfected with 75% ethanol, and a hole was carefully drilled. Subsequently, 10 *μ*l of Walker 256 cells (1 × 10^6^ cells) or heat-killed cells (sham group) were slowly injected into the bone marrow canal using a 25 *μ*l microsyringe. The cells were then allowed to fill the bone cavity for 1 min to equalize the pressure. After withdrawing the needle, the injection site was sealed with bone wax to prevent tumor cells from leaking beyond the bone injection site. Finally, the sterile incision was sutured with fibrin glue.

### 2.4. Intrathecal (i.t.) Catheterization

According to previous studies [38, 39], a PE-10 (Kang Mei Biological Co., China) microtube was inserted into the intervertebral space between L4 and L5 for drug delivery to reduce systemic effects on bone tumors. Briefly, rats were anesthetized via intraperitoneal injection with sodium pentobarbital (50 mg/kg), their backs were shaved, and then a polyethylene tubing (PE-10) was inserted into the disc and extended into the enlarged subarachnoid space in the lumbar region. The microtubules were secured to the adjacent ligaments with 3-0 sutures, and the surgical incision was then sutured. To confirm the success of catheterization, 10 *μ*l of lidocaine (2%) was injected through the catheter the next day. Only the rats that showed complete paralysis of both hind limbs after lidocaine administration was used in subsequent experiments.

### 2.5. Drugs and Administration

Rosiglitazone (PPAR-*γ* agonist, Cat. No. ab120762, Abcam, UK) was dissolved in 5% dimethyl sulfoxide (DMSO, Cat. No. D2650, Sigma-Aldrich, USA) in sterile saline. GW9662 (PPAR-*γ* antagonist, Cat. No. HY-16578, MedChemExpress, USA) was dissolved in 5% DMSO (Cat. No. D2650, Sigma-Aldrich, USA) and 20% Tween 80 (Cat. No. HY-Y1891, MedChemExpress, USA) in sterile saline. Pyrrolidinedithiocarbamate ammonium (PDTC, a selective NF-*κ*B inhibitor, Cat. No. HY-18738, MedChemExpress, USA) was dissolved in saline. MCC950 sodium (a selective NLRP3 inhibitor, Cat. No. HY-12815A, MedChemExpress, USA) was dissolved in saline. The drug administration protocol was as follows: rosiglitazone (50, 100, and 200 *μ*g/10 *μ*l), GW9662 (10 mg/kg), PDTC (120 *μ*g/10 *μ*l), and MCC950 (1 mg/kg) were administrated from day 14 to day 18 once daily after BCP model establishment using an intrathecal catheter to minimize the systemic effects on tumor growth.

### 2.6. Cell Culture

According to previous study [40], Walker 256 cells were cultured in RPMI 1640 medium (GIBCO; MD, USA) containing 10% fetal bovine serum (FBS, heat-inactivated) (HyClone; Utah, USA), 1% L-glutamine, and 2% penicillin/streptomycin (GIBCO; MD, USA) and incubated at 37°C and 5% CO_2_.

### 2.7. CCK8 Assay

To assess whether rosiglitazone affects tumor cell proliferation, a CCK8 assay was performed. Walker 256 cells (1 × 10^4^ cells) were plated into each well of a 96-well flat-bottom plate in a final volume of 100 *μ*l/well. Rosiglitazone was added to the cells at concentrations of 0 *μ*M, 25 *μ*M, 50 *μ*M, 100 *μ*M, and 200 *μ*M and then incubated at 37°C for 3 h and 6 h. At the end of the incubation period, 10 *μ*l of CCK8 assay solution (Dojindo, Japan) was added to each well containing control samples and samples containing rosiglitazone. Following the procedure from a previous study [40], samples were kept in an incubator for 60 min, after which the absorbance (OD) was measured at 450 nm using a Multiskan GO spectrophotometer (USA). Cell viability (% of control) was determined using the following equation: (mean absorbance of treated cells/mean absorbance of control cells) × 100.

### 2.8. Rotarod Testing

Motor dysfunction can substantially affect the results of nociceptive behavior tests; therefore, a rotation test was used to evaluate whether i.t. injection of different doses of rosiglitazone would affect the motor function of rats with BCP. As described previously [36], all rats were trained for at least 3 min per day for at least 5 days prior to baseline testing. They were placed on a 70 mm diameter rotating tripod (Ugo Basile, Varese, Italy), followed by linear acceleration from 4 to 40 rpm over 3 min. The rats were placed on a rotating pole, and the time spent on the pole was measured at the 3-minute cut-off time. The results were expressed as percentages of the baseline values for each group.

### 2.9. Mechanical Allodynia Test

Paw withdrawal threshold (PWT) in response to von Frey filament (BME-404; Institute of Biological Medicine, Academy of Medical Science, Beijing, China) stimulation was measured to represent mechanical allodynia. In brief, the rats were placed in a single transparent Plexiglas compartment on a metal mesh floor (grid: 0.5 cm × 0.5 cm; compartment: 10 cm × 10 cm × 15 cm) and allowed to acclimatize for a minimum of 30 min before performing the experiment. The values were averaged to yield the PWT after five consecutive tests at 10 s intervals. PWT was measured in rats before surgery and on days 3, 6, 12, or 18 after surgery, as well as at various time points after intrathecal injection. All behavioral testing procedures were performed by researchers who were blinded to treatment group.

### 2.10. CATWALK Automated Gait Analysis

Gait analysis was performed using the CATWALK XT system (AsterWee Information Technology), which records and analyzes the voluntary movements of rats on a closed walkway and has proven to be a reliable method for measuring pain-related behavior [41–43]. In brief, green light was reflected from the inside onto a glass panel that held an enclosed corridor with a red backlight above it. The camera was installed under the device, and the paw prints were recorded using green light when the rat's paw was in contact with the glass plate while walking along the corridor. When the rat walked across the glass floor, the high-speed camera located under the instrument captured the image of the illuminated area of each paw and transmitted the data to the gait analysis software (CATWALK XT, AsterWee Information Technology). A complete passage through the tunnel was collected as valid data. The previous studies [44–46] showed that the following parameters have been the most acknowledged and applied in pain models: area data (CatWalk-max contact area) and intensity data (CatWalk-mean intensity). In this study, three available parameters were identified to evaluate dynamic behaviors related to BCP: (1) “max contact area” is the print area during maximum hind paw contact; (2) “max contact max intensity” is the maximum intensity during maximum hind paw contact; and (3) “mean intensity” is the average of the hind paw intensity at all time points. To clearly illustrate the alteration of the intensity and area data for the ipsilateral (left) hind paw, we used the formula left hind paw/right hind paw (LH/RH) to remove the influence of confounding factors. Data were calculated as the percentage of the ipsilateral (left)/contralateral (right) hind paws.

### 2.11. Histochemical Staining

To observe the extent of tumor invasion and bone destruction, rats were euthanized via intraperitoneal injection using an excessive amount of sodium pentobarbital (100 mg/kg) on the 18^th^ day after surgery. Tissues from the left tibia around the inoculation site (a total of 1 cm) were collected. The tibia bones were fixed with 4% paraformaldehyde and decalcified in a 10% ethylenediaminetetraacetic acid mixture for 24 h, embedded in paraffin, and cut into 8 *μ*m thick sections. All images were acquired using a 20x or 40x objective using a microscope (Olympus BX 51, Japan). Immunohistochemical analysis was performed by three well-trained pathologists who were blinded to the treatment during the analysis.

### 2.12. Three-Dimensional (3D) CT Reconstruction

Three-dimensional CT bone reconstruction technology was used to observe bone destruction. Before receiving CT tomography, the rats were anesthetized via intraperitoneal injection with sodium pentobarbital (50 mg/kg). According to a previous study [37], we performed analysis by acquiring images using the following parameters: helical scanning at 120 kVp, care dose 4D technique, layer thickness of 1 mm, layer interval of 1 mm, and kemel of U30u medium smooth, with CT imaging of SD rat tibia at a high resolution (FOV 100 mm). All images were processed and analyzed using a Siemens Syngo MMWP postprocessing workstation.

### 2.13. Western Blot

After measuring the PWT, the rats were deeply anesthetized via intraperitoneal injection with sodium pentobarbital (100 mg/kg). The L4-L5 segments of the spinal cord were harvested and stored in a deep freezer (-80°C). Tissues were homogenized in RIPA lysis buffer containing a protease inhibitor PMSF. The homogenates were centrifuged at 12,000 rpm for 15 min at 4°C. Then, the supernatant was collected, protein concentration was measured using a BCA protein assay (Pierce), and the proteins were denatured in loading buffer for 10 min at 95°C. Lysates (total protein, 40 *μ*g) were separated via electrophoresis in 7.5% sodium dodecyl sulfate-polyacrylamide gels and then transferred onto polyvinylidene fluoride membranes (PVDF). Membranes were blocked with 5% skim milk for 2 h at RT and incubated overnight at 4°C with rabbit anti-PPAR-*γ* antibody, (Cat. No. 16643–1-AP, Proteintech, 1 : 1000), rabbit anti-NLRP3 antibody, (Cat. No. DF7438, Affinity, 1 : 1000), rabbit anti–p-NF-*κ*B p65 antibody (Cat. No. AF2006, Affinity, 1 : 1000), and rabbit anti-*β*-actin (Cat. No. AF7018, Affinity, 1 : 2000). The membranes were washed with Tris-buffered saline containing Tween-20 and then incubated with horseradish peroxidase labeled goat anti-rabbit secondary antibody (Jackson, 1 : 2000) for 2 h at RT. The immunoreactive bands were detected using enhanced chemiluminescence (Thermo Scientific) and exposed to X-ray films. Band intensities were quantified using Image Lab software (Bio-Rad Laboratories). Results were normalized to the loading control *β*-actin and expressed as fold of the control.

### 2.14. Immunofluorescence Staining

Rats were deeply anesthetized via intraperitoneal injection with sodium pentobarbital (100 mg/kg), and then an intracardiac injection of 4% paraformaldehyde was administered. The L3-L5 segments of the spinal cord were removed, fixed in 4% paraformaldehyde for 6 h, and dehydrated in a gradient sucrose solution (15%-30%) at 4°C for 48 h. These spinal cord samples were embedded in OCT compound (Sakura, USA) at -20°C and then cut into 20 *μ*m thick sections. The sections were infiltrated with 0.2% TritonX-100 for 15 min and then blocked with 5% bovine serum albumin for 1 h at RT. Subsequently, the sections were incubated overnight at 4°C with different antibodies, PPAR-*γ* (Cat. No. 16643–1-AP, rabbit source, Proteintech, 1 : 50), NLRP3 (Cat. No. DF7438, rabbit source, Affinity, 1 : 50), p-NF-*κ*B p65 (Cat. No. sc-166748, mouse source, Santa, 1 : 100), IBA-1 (ionized calcium binding adapter molecule 1, microglia marker, Cat. No. ab48004, goat source, Abcam, 1 : 200), and IBA-1 (microglia marker, Cat. No. #17198, rabbit source, CST, 1 : 100), NeuN (neuronal nuclei, neuronal marker, Cat. No. ab279297, rabbit source, Abcam, 1 : 100), NeuN (neuronal marker, Cat. No. ab104224, mouse source, Abcam, 1 : 100), GFAP (glial fibrillary acidic protein, astrocyte marker, Cat. No. #80788, rabbit source, CST, 1 : 100), and GFAP (astrocyte marker, Cat. No. C9205, mouse source, Sigma-Aldrich, 1 : 300). Subsequently, the sections were incubated for 1 h at room temperature with Alexa Fluor-488 (Cat. No. ab150073, donkey anti-rabbit, Abcam, 1 : 500) or Alexa Fluor-488 (Cat. No. ab150105, donkey anti-mouse, Abcam, 1 : 500) or Alexa Fluor-488 (Cat. No. ab150129, donkey anti-goat, Abcam, 1 : 500,) or Alexa Fluor-594 (Cat. No. ab150076, donkey anti-rabbit, Abcam, 1 : 500) or Alexa Fluor-594 (Cat. No: ab150108, donkey anti-mouse, Abcam, 1 : 500) secondary antibodies, and nuclei were stained with 1 *μ*g/ml DAPI (H-1200 VECTAS HIELD Antifade Mounting Medium containing DAPI). Images were captured using a multiphoton confocal microscope (Leica Microsystems, Wetzlar, Germany).

### 2.15. Statistical Analysis

GraphPad Prism version 6.0 for Windows (San Diego, CA, USA) was used to conduct all statistical analyses. No statistical power calculation was conducted before the study. The sample sizes were based on our previous knowledge and research. Nociceptive behavioral tests, rotarod tests, and CCK-8 assays over time among groups were tested using two-way repeated-measures analysis of variance (ANOVA) followed by Bonferroni's *post hoc* tests. Differences in western blot and CATWALK gait results for each group were tested using one-way ANOVA with a Student-Newman-Keuls *post hoc* test. All data are presented as the mean ± standard deviation, and *p* < 0.05 was considered statistically significant. The investigator who performed the analyses was blinded to the experimental design.

## 3. Results

### 3.1. Intramedullary Inoculation of Walker 256 Cells Induces Pain Hypersensitivity and the Destruction of Cortical Bone in the Rat Tibia

In this study, a rat model of BCP was established by inoculating of Walker 256 breast cancer cells into the medullary cavity of the tibia, which was validated using four methods: von-Frey, CT 3D imaging, hematoxylin staining, and CATWALK gait analysis.

**Figure 1:**
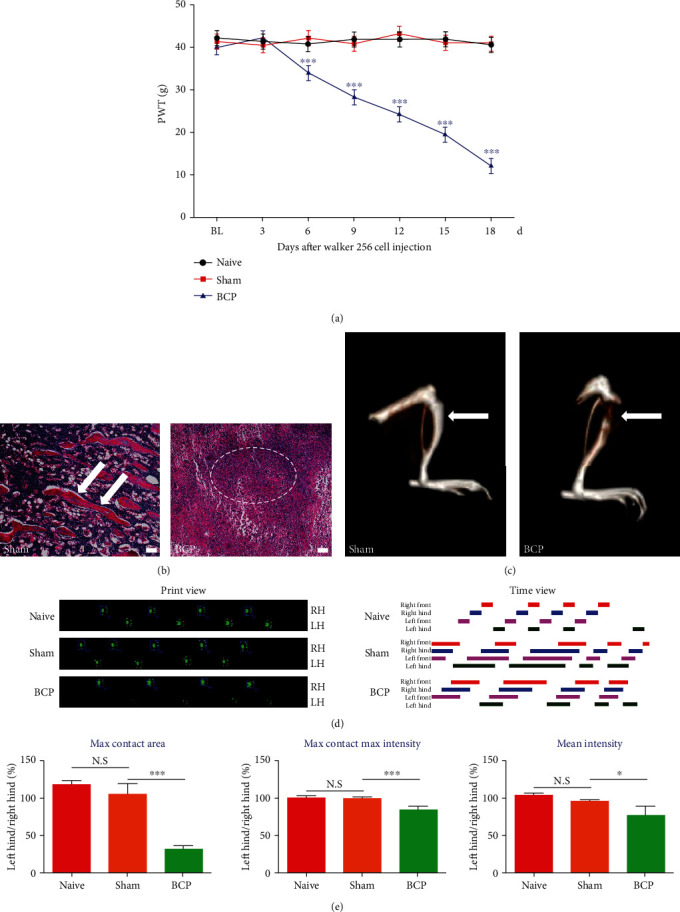
(a) A rat model of bone cancer pain (BCP) was successfully established. The mechanical pain threshold of the ipsilateral (left) hind foot in BCP rats showed significantly decrease from day 6 to day 18 after surgery (^∗∗∗^*p* < 0.001 vs. the sham group; *n* = 6, two-way repeated measures ANOVA (a)). (b) Hematoxylin-eosin staining showed that the normal bone trabecular structure (arrows) was destroyed in the bone marrow cavity of BCP rats accompanied by cancer cell infiltration after inoculation (within the dotted lines) (*n* = 10). Scale bars: 200 *μ*m. *n* represents the number of experimental animals in each group. (c) Three-dimensional CT reconstruction showed evident cortical bone destruction in the tibial bone (arrows) 18 days after cancer cell inoculation. (d) Representative CATWALK gait results, including print view and timing view in the naïve, sham, and BCP groups. Print area is the size of the paw print. Print time represents the related contact time. (e) The percentages of left/right hind paw ratio of maximum contact area, maximum contact maximum intensity, and mean intensity decreased significantly after cancer cell inoculation. Max contact area is the print area during maximum hind paw contact; max contact max intensity is the maximum intensity during maximum hind paw contact; and mean intensity is the average of the hind paw intensity at all time points. All data were calculated as the percentage of the ipsilateral (left)/contralateral (right) hind paw (^∗^*p* < 0.05 and ^∗∗∗^*p* < 0.001 vs. the sham group; *n* = 6, one-way ANOVA (e)). LH: left hind paw; RH: right hind paw; BCP: bone cancer pain. N.S: not statistically significant.

We investigated the chronic pain state induced by the BCP model and assessed mechanical allodynia in the ipsilateral (left) hind paw. Compared with the sham group, the rats in the BCP group showed evident mechanical hyperalgesia with the progression of bone cancer from the sixth day after the operation (^∗∗∗^*p* < 0.001 vs. the sham group; *n* = 6, two-way repeated measures ANOVA, Figure 1(a)). There were no statistically significant differences in PWT between the naive and sham groups (*p* > 0.05 vs. naive group; *n* = 6, two-way repeated measures ANOVA, Figure 1(a)). To further verify the validation of the BCP model, radiological imaging of the rat tibia was performed to assess bone destruction 18 days after cancer cell inoculation. Reconstructed 3D CT images showed substantia bone destruction in the tibia of rats with BCP, suggesting the development of bone cancer in the tibia (Figure 1(c)). No radiological changes were observed in control animals treated with heat-killed tumor cells (Figure 1(c)). Histopathological analysis was performed on rats 18 days after tumor cell inoculation, and the occurrence of bone cancer was confirmed. Hematoxylin-eosin staining showed normal trabecular bone structure (arrows) in the bone marrow cavity of rats in the sham group (Figure 1(b)). In contrast, the bone trabecular structure in the bone marrow cavity of the BCP group disappeared and was accompanied by a large amount of cancer cell infiltration (dotted lines) (Figure 1(b)).

CATWALK gait analysis is considered a valuable method for objectively assessing chronic pain behavior in several neuropathic and inflammatory pain models. Here, we applied CATWALK analysis in the context of BCP. We selected the following parameters that were significantly altered in SD rats: (1) maximum contact area, (2) maximum contact maximum intensity, and (3) mean intensity. We found that the percentages of left/right hind paw ratio in maximum contact area, maximum contact maximum intensity, and mean footprint intensity were approximately 100% in sham rats on day 18. After tumor inoculation, these three parameters decreased significantly, which could be representative of BCP behaviors from different aspects (^∗^*p* < 0.05 and ^∗∗∗^*p* < 0.001 vs. the sham group; *n* = 6, one-way ANOVA, Figure 1(e)).

### 3.2. Effect of Rosiglitazone on the Growth of Tumor Cells and on Motor Function in Rats with BCP

**Figure 2:**
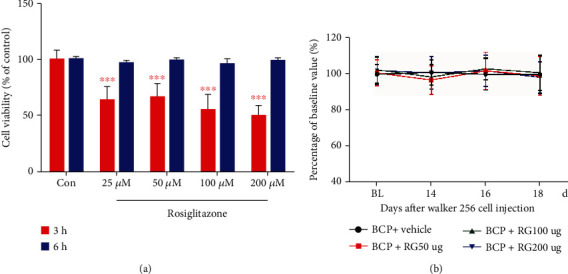
Effect of rosiglitazone on the growth of tumor cells and the effect of repeated intrathecal administration of rosiglitazone determined through the rotarod test. (a) Compared with the control group, rosiglitazone significantly inhibited the viability of Walker 256 cells at 3 h (^∗∗∗^*p* < 0.001 vs. controls; two-way repeated measures ANOVA (a)), with no significant difference at 6 h (*p* > 0.05 vs. controls; two-way repeated measures ANOVA (a)). (b) Compared with vehicle treatment bone cancer pain (BCP) group, intrathecal injection of rosiglitazone (50, 100, and 200 *μ*g) for 5 days did not affect the motor performance of rats with BCP (*p* > 0.05 vs. vehicle control group; two-way repeated measures ANOVA (b)). The results are expressed as the percentage of each rat's baseline value. BL: baseline; Con: control; RG: rosiglitazone.

To investigate whether rosiglitazone affects the growth of tumor cells, we tested the viability of Walker 256 cells after treatment with different concentrations (0 *μ*M, 25 *μ*M, 50 *μ*M, 100 *μ*M, and 200 *μ*M) of rosiglitazone for 3 h and 6 h. The results showed that compared to the tumor cells of the control group, rosiglitazone significantly inhibited the viability of Walker 256 cells at 3 h (^∗∗∗^*p* < 0.001 vs. controls; two-way repeated measures ANOVA, Figure 2(a)), whereas there was no significant difference in the viability between the cells of the rosiglitazone-treated and control groups at 6 h (*p* > 0.05 vs. controls; two-way repeated measures ANOVA, Figure 2(a)). Therefore, to avoid the effect of intraperitoneal administration of rosiglitazone on tumor growth, we selected intrathecal administration in subsequent experiments.

Motor dysfunction demonstrated evident effects on the results of nociceptive behavior determination; therefore, it was essential to explore whether different dosages of rosiglitazone (50, 100, and 200 *μ*g) could induce an impairment of motor functions in rats with BCP. A rotarod test was performed to assess the influence of repeated administration of rosiglitazone on motor function. As illustrated in Figure 2(b), there was no difference observed in the performance of rats in the vehicle control group or in the rosiglitazone treatment (50, 100, and 200 *μ*g) groups, indicating that repeated intrathecal administration of rosiglitazone demonstrated no evident measurable impact on motor function (*p* > 0.05 vs. the vehicle control group; two-way repeated measures ANOVA, Figure 2(b)).

### 3.3. Rosiglitazone Attenuated BCP by Activating PPAR-*γ*

**Figure 3:**
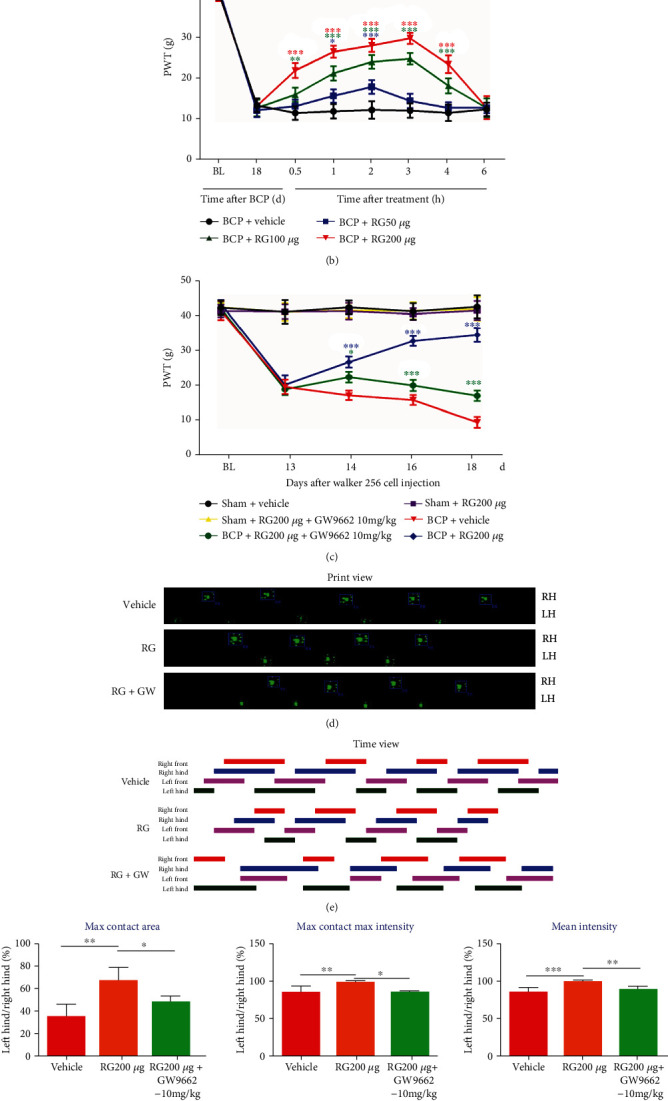
Rosiglitazone attenuated bone cancer pain (BCP) by activating proliferator-activated receptor-*γ* (PPAR-*γ*). (a) Compared to that of rats in the vehicle-treated BCP group, repeated intrathecal injection of rosiglitazone at 50, 100, and 200 *μ*g significantly increased the paw withdrawal threshold (PWT) of BCP rats in a dose-dependent manner from postoperative days (POD) 14–18 (^∗^*p* < 0.05 and ^∗∗∗^*p* < 0.001 vs. the vehicle control group; *n* = 6, two-way repeated measures ANOVA (a)). (b) A single dose of rosiglitazone (50, 100, and 200 *μ*g) reversed mechanical hypersensitivity in the later stages of BCP in a dose-dependent manner (^∗^*p* < 0.05, ^∗∗^*p* < 0.01, and ^∗∗∗^*p* < 0.001 vs. the vehicle control group; *n* = 6, two-way repeated measures ANOVA (b)). (c) The PPAR-*γ* antagonist GW9662 reversed the analgesic effect of rosiglitazone in BCP rats (^∗^*p* < 0.05 and ^∗∗∗^*p* < 0.001 vs. the BCP+RG group; *n* = 6, two-way repeated measures ANOVA (c)). Rosiglitazone and GW9662 treatment did not affect the basal pain threshold of sham rats (*p* > 0.05 vs. the vehicle control group; two-way repeated measures ANOVA (c)). (d, e) Representative CATWALK gait analysis results, including print view and timing view in the BCP, BCP+rosiglitazone, and BCP+rosiglitazone+GW9662 groups. Print area is the size of the paw print. Print time represents the related contact time. (f) Compared with those in the BCP group, the percentages of left/right hind paw ratio of maximum contact area, maximum contact maximum intensity, and mean intensity were significantly increased in the group treated with 200 *μ*g rosiglitazone (^∗∗^*p* < 0.01 and ^∗∗∗^*p* < 0.001 vs. vehicle control group; *n* = 6, one-way ANOVA (f)), which was reversed upon treatment with GW9662 at a dose of 10 mg/kg (^∗^*p* < 0.05 and ^∗∗^*p* < 0.01 vs. the BCP+RG group; *n* = 6, one-way ANOVA (f)). Max contact area is the print area during maximum hind paw contact; max contact max intensity is the maximum intensity during maximum hind paw contact; and mean intensity is the average of the hind paw intensity at all time points. All data were calculated as the percentage of the ipsilateral (left)/contralateral (right) hind paw. (g) Western blotting results showed that compared with the vehicle treatment group, rosiglitazone treatment remarkably increased the expression of PPAR-*γ* in the spine of BCP rats (^∗∗^*p* < 0.01 vs. the vehicle control group; *n* = 4, one-way ANOVA (g)), which was reversed by preadministration of GW9662 (^∗∗^*p* < 0.01 vs. the BCP+RG group; *n* = 4, one-way ANOVA (g)). BL: baseline; GW: GW9662; RG: rosiglitazone. N.S: not statistically significant.

To evaluate whether repeated intrathecal administration of rosiglitazone could alleviate BCP in rats, different doses (50, 100, and 200 *μ*g) of rosiglitazone were intrathecally injected once daily for 5 days from postoperative days (POD) 14–18 and behavioral tests were performed from POD 13–18. Compared with the control group, repeated injection of rosiglitazone at 50, 100, and 200 *μ*g significantly increased the PWT of BCP rats in a dose-dependent manner indicating that rosiglitazone could attenuate established BCP (^∗^*p* < 0.05 and ^∗∗∗^*p* < 0.001 vs. the vehicle control group; *n* = 6, two-way repeated measures ANOVA, Figure 3(a)).

Next, to study whether rosiglitazone affects mechanical hyperalgesia in BCP rats in the late stage of bone cancer, single doses of rosiglitazone (50, 100, and 200 *μ*g,) were injected intrathecally on POD 18. Behavioral tests were performed 1 h before and 0.5, 1, 2, 3, 4, and 6 h after rosiglitazone administration. As shown in Figure 3(b), compared with the vehicle treatment group, PWT was significantly augmented at 0.5 h until 4 h after administration in the BCP group treated with 100 and 200 *μ*g rosiglitazone. However, in the BCP group treated with a low dose of rosiglitazone (50 *μ*g), a certain analgesic effect was only observed at 1 h and 2 h after administration (^∗^*p* < 0.05, ^∗∗^*p* < 0.01, and ^∗∗∗^*p* < 0.001 vs. the vehicle control group; *n* = 6, two-way repeated measures ANOVA, Figure 3(b)).

To determine whether rosiglitazone attenuated BCP via activation of PPAR-*γ*, the PPAR-*γ* antagonist GW9662 (10 mg/kg) was injected intrathecally 30 min before the administration of rosiglitazone (200 *μ*g) once daily for 5 days from POD 14–18. Behavioral tests were performed on POD 13–18. As shown in Figure 3(c), the analgesic effect of rosiglitazone on BCP was significantly reversed by GW9662 treatment (^∗^*p* < 0.05 and ^∗∗∗^*p* < 0.001 vs. the BCP+RG group; *n* = 6, two-way repeated measures ANOVA, Figure 3(c)). Interestingly, we found that intrathecal injection of rosiglitazone and GW9662 did not change the PWT of rats in the sham group (*p* > 0.05 vs. the vehicle control group; two-way repeated measures ANOVA, Figure 3(c)). These results indicate that rosiglitazone ameliorates BCP by activating PPAR-*γ*.

The results of CATWALK gait analysis also confirmed that rosiglitazone relieves BCP by activating PPAR-*γ*. Behavioral tests were performed on POD18 and 3 h after rosiglitazone injection. As illustrated in Figures 3(d)–3(f), repeated intrathecal injection of rosiglitazone partially, but significantly, reversed BCP-induced gait alterations (^∗∗^*p* < 0.01 and ^∗∗∗^*p* < 0.001 vs. the vehicle control group; *n* = 6, one-way ANOVA, Figure 3(f)). However, repetitive intrathecal administration of GW9662 significantly reversed the gait changes caused by rosiglitazone (^∗^*p* < 0.05 and ^∗∗^*p* < 0.01 vs. the BCP+RG group; *n* = 6, one-way ANOVA, Figure 3(f)).

Subsequent molecular experiments further confirmed that rosiglitazone alleviated BCP by activating PPAR-*γ*. Western blotting results showed that rosiglitazone treatment significantly increased PPAR-*γ* expression in the spinal cord of BCP rats compared to the vehicle treatment group (^∗∗^*p* < 0.01 vs. the vehicle control group; *n* = 4, one-way ANOVA, Figure 3(g)), which was reversed by preadministration with GW9662 (^∗∗^*p* < 0.01 vs. the BCP+RG group; *n* = 4, one-way ANOVA, Figure 3(g)). In summary, it was confirmed that rosiglitazone alleviates BCP by activating PPAR-*γ*.

### 3.4. Endogenous Expression and Cellular Localization of Spinal PPAR-*γ*

**Figure 4:**
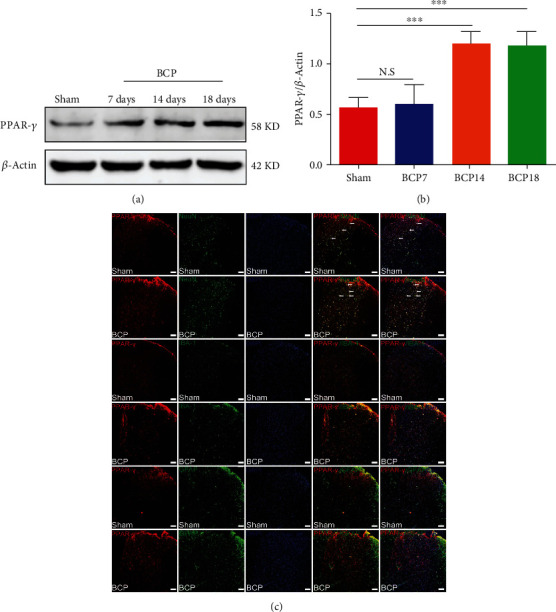
Endogenous expression and cellular localization of proliferator-activated receptor-*γ* (PPAR-*γ*). (a, b) Western blot results revealed a significant increase in PPAR-*γ* expression at 14 and 18 days in bone cancer pain (BCP) rats (^∗∗∗^*p* < 0.001 vs. the sham group; *n* = 4, one-way ANOVA (a, b)). There was no difference in the expression of PPAR-*γ* between rats in the sham group and BCP rats at 7days (*p* > 0.05 vs. sham group; *n* = 4, one-way ANOVA (a, b)). (c) Immunofluorescence results showed that in the dorsal horn of the spinal cord of BCP rats, PPAR-*γ* (red) was primarily expressed in neurons (green) rather than the astrocytes (green) or microglia (green). Lumbar enlargements were collected on day 18 after the operation or cancer cell inoculation. Sections were counterstained with DAPI (blue) to label cell nuclei. The white arrows indicate colocalization of PPAR-*γ* with NeuN (neuronal nuclei, neuronal-specific marker), GFAP (glial fibrillary acidic protein, astrocyte-specific marker), and IBA-1- (ionized calcium binding adapter molecule 1, microglial specific marker) immunoreactive cells in the spinal dorsal horn, respectively; *n* = 4. Scale bar = 50 *μ*m. *n* represents the number of experimental animals in each group. N.S: not statistically significant.

At 7, 14, and 18 days after the successful establishment of the BCP model, the lumbar spinal cord enlargements (L3-L5) were collected. Western blotting and immunofluorescence assays were performed to detect changes in the endogenous expression and cellular localization of PPAR-*γ*. Western blot results revealed a significant increase in PPAR-*γ* levels at POD 14 and 18 in BCP rats (^∗∗∗^*p* < 0.001 vs. the sham group; *n* = 4, one-way ANOVA, Figures 4(a) and 4(b)), whereas there was no difference between rats in the sham group and rats at POD 7 (*p* > 0.05 vs. the sham group; *n* = 4, one-way ANOVA, Figures 4(a) and 4(b)). To define the cellular localization of PPAR-*γ* in the spinal cord dorsal horn, triple staining of PPAR-*γ* with three different cell markers (NeuN, IBA-1, and GFAP), and DAPI was performed. Results showed that PPAR-*γ* colocalized primarily with neurons, but not with microglia and astrocytes, in BCP rats (Figure 4(c)).

### 3.5. Rosiglitazone Attenuates BCP by Activating PPAR-*γ* to Inhibit the NF-*κ*B/NLRP3 Inflammatory Axis in Spinal Cord Neurons

The aforementioned results demonstrated that rosiglitazone exerts an evident analgesic effect on BCP through the activation of PPAR-*γ*. Previous studies have suggested that rosiglitazone exerts its anti-inflammatory and neuroprotective effects by inhibiting the NLRP3 inflammasome and reducing the expression of NF-*κ*B. Therefore, we investigated the effect of rosiglitazone on the NF-*κ*B/NLRP3 inflammatory axis in BCP rats.

**Figure 5:**
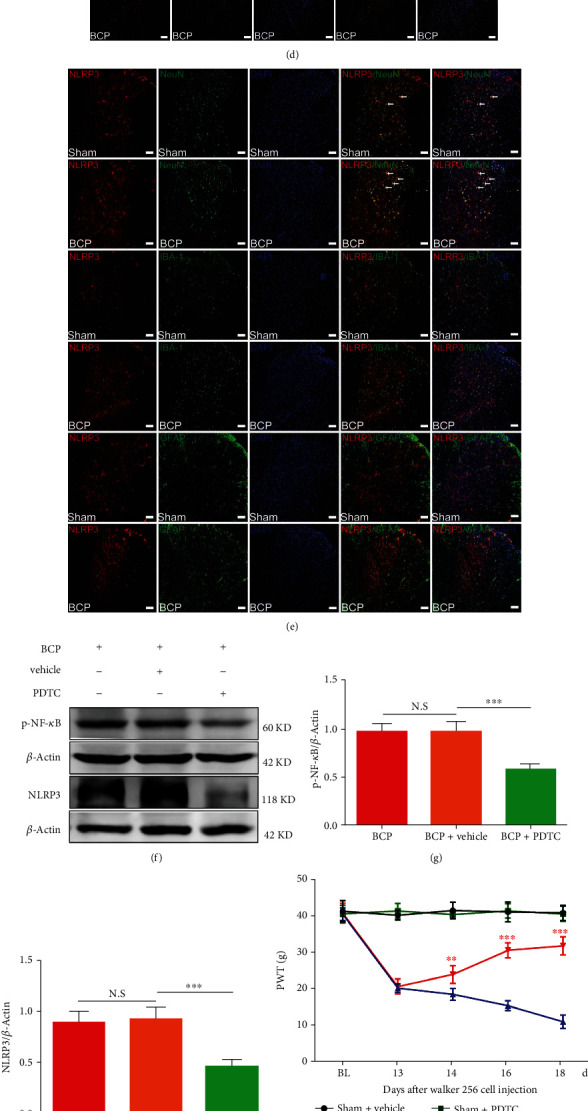
Rosiglitazone attenuates bone cancer pain (BCP) through the activation of proliferator-activated receptor-*γ* (PPAR-*γ*) to inhibit the nuclear factor-kappa B (NF-*κ*B)/nod-like receptor protein 3 (NLRP3) inflammatory axis in spinal cord neurons. (a–c) Tumor cell inoculation significantly induced the activation of NF-*κ*B and NLRP3 in the spinal cord of BCP rats (^∗∗∗^*p* < 0.001 vs. the sham group; *n* = 4, one-way ANOVA (a–c)). (d, e) Immunofluorescence results showed that in the dorsal horn of the spinal cord of BCP rats, p-NF-*κ*B (red) and NLRP3 (red) were primarily expressed in neurons (green) rather than astrocytes (green) or microglia (green). Lumbar enlargements were collected on day 18 after the operation or cancer cell inoculation. Sections were counterstained with DAPI (blue) to label cell nuclei. The white arrows indicate colocalization of p-NF-*κ*B and NLRP3 with NeuN (neuronal nuclei, neuronal-specific marker), GFAP (glial fibrillary acidic protein, astrocyte specific marker), and IBA-1- (ionized calcium binding adapter molecule 1, microglial specific marker) immunoreactive cells in the spinal dorsal horn, respectively; *n* = 4. Scale bar = 50 *μ*m. *n* represents the number of experimental animals in each group. (f–i) Repeated intrathecal injection with PDTC, an NF-*κ*B inhibitor, significantly inhibited the established BCP (^∗∗^*p* < 0.01 and ^∗∗∗^*p* < 0.001 vs. the vehicle control group; *n* = 6, two-way repeated measures ANOVA (i)) and BCP-induced NF-*κ*B/NLRP3 activation (^∗∗∗^*p* < 0.001 vs. the vehicle control group; *n* = 4, one-way ANOVA (f–h)). (j–l) Repeated intrathecal injection with MCC950, an NLRP3 inhibitor, significantly inhibited the established BCP (^∗∗^*p* < 0.01 and ^∗∗∗^*p* < 0.001 vs. the vehicle control group; *n* = 6, two-way repeated measures ANOVA (l)) and BCP-induced NLRP3 activation (^∗∗∗^*p* < 0.001 vs. the vehicle control group; *n* = 4, one-way ANOVA (j, k)). (m–o) Rosiglitazone inhibits the BCP-induced activation of the NF-*κ*B/NLRP3 inflammatory axis, whereas GW9662 reversed this effect (^∗^*p* < 0.05 and ^∗∗∗^*p* < 0.001 vs. the vehicle control group; ^∗^*p* < 0.05 and ^∗∗∗^*p* < 0.001 vs. the BCP+RG group; *n* = 4, one-way ANOVA (m–o)). N.S: not statistically significant.

Similar to the detection of PPAR-*γ*, we euthanized BCP rats at 7, 14, and 18 days after surgery to obtain their spinal cord lumbar enlargements for western blot and immunofluorescence analysis. As depicted in Figures 5(a)–5(c), western blotting results showed that compared with the sham group, the expression of p-NF-*κ*B and NLRP3 in the spinal cord of BCP rats increased significantly with bone cancer progression (^∗∗∗^*p* < 0.001 vs. the sham group; *n* = 4, one-way ANOVA, Figures 5(a)–5(c)). Subsequent immunofluorescence results also confirmed this and showed that p-NF-*κ*B and NLRP3 colocalized primarily with neurons but not with microglia and astrocytes in sham group rats and BCP rats (Figures 5(d) and 5(e)).

To further elucidate the relationship between NF-*κ*B, NLRP3, and BCP, PDTC (120 *μ*g), an antagonist of NF-*κ*B, and MCC950 (1 mg/kg), an antagonist of NLRP3, were administered as intrathecal injections once daily for 5 days from POD 14–18. Behavioral tests were performed on POD13-18. The results showed that PDTC (120 *μ*g) significantly reversed the established mechanical hyperalgesia in BCP rats, as did MCC950 (1 mg/kg) (^∗∗^*p* < 0.01 and ^∗∗∗^*p* < 0.001 vs. the vehicle control group; *n* = 6, two-way repeated measures ANOVA, Figures 5(i) and 5(l)); however, neither PDTC nor MCC950 treatment changed the PWT in sham group rats (*p* > 0.05 vs. the vehicle control group; two-way repeated measures ANOVA, Figures 5(i) and 5(l)). The results of subsequent molecular experiments showed that compared with the vehicle treatment group, the expression of p-NF-*κ*B in the PDTC group and NLRP3 in the MCC950 group was reduced (^∗∗∗^*p* < 0.001 vs. the vehicle control group; *n* = 4, one-way ANOVA, Figure 5(f), 5(g), and 5(j)–5(k)). In addition, repeated intrathecal injections of PDTC also remarkably inhibited BCP-induced NLRP3 activation in the rat spinal cord (^∗∗∗^*p* < 0.001 vs. the vehicle control group; *n* = 4, one-way ANOVA, Figures 5(f) and 5(h)), which demonstrated that tumor inoculation induced NF-*κ*B/NLRP3 inflammatory axis activation in the spinal cord of BCP rats.

Finally, we explored the effect of rosiglitazone on the BCP-induced NF-*κ*B/NLRP3 inflammatory axis in the rat spinal cord. The PPAR-*γ* antagonist GW9662 (10 mg/kg) was injected intrathecally 30 min before the administration of rosiglitazone (200 *μ*g) once daily for 5 days on POD 14–18. Rats were euthanized on POD 18 to obtain their spinal cords for subsequent molecular experiments. Western blotting results showed that repeated intrathecal treatment with rosiglitazone significantly inhibited the BCP-induced activation of the NF-*κ*B/NLRP3 inflammatory axis in the spinal cord, whereas GW9662 treatment reversed this effect (^∗^*p* < 0.05 and ^∗∗∗^*p* < 0.001 vs. the vehicle control group; ^∗^*p* < 0.05 and ^∗∗∗^*p* < 0.001 vs. the BCP+RG group; *n* = 4, one-way ANOVA, Figures 5(m)–5(o)). These results suggest that rosiglitazone inhibits the NF-*κ*B/NLRP3 inflammatory axis of spinal cord neurons by activating PPAR-*γ*, thereby attenuating BCP.

## 4. Discussion

Currently, cancer pain seriously affects the quality of life of bone cancer patients due to the lack of effective treatments. In this study, we investigated the antinociceptive effects and possible mechanisms of action of rosiglitazone in a rat model of BCP. Our main findings are as follows: (1) rosiglitazone attenuated established BCP by activating PPAR-*γ* in the spinal cord, which was reversed by the PPAR-*γ* antagonist GW9662; (2) rosiglitazone increased PPAR-*γ* expression in the spinal cord neurons of BCP rats; (3) tumor cell inoculation induces the activation of the NF-*κ*B/NLRP3 inflammatory axis in the spinal cord dorsal horn neurons of BCP rats; and (4) rosiglitazone inhibited the NF-*κ*B/NLRP3 inflammatory axis in spinal cord neurons by activating PPAR-*γ* to attenuate BCP. These findings provide evidence for a new potential target in the treatment and management of BCP.

The Walker 256 mammary cell-induced rat model of BCP has been widely used to study the mechanism of analgesic drugs because of its stable and persistent pain-related behavior. In this study, the mechanical withdrawal threshold showed a significant decrease on day 6 and continued to decrease until the end of the experiment on day 18. Hematoxylin-eosin staining showed cancer cell infiltration in the bone marrow cavity of BCP rats. Three-dimensional CT reconstruction showed significant cortical bone destruction in the tibia after tumor inoculation, which was consistent with our previous findings [33, 36]. In addition, we applied CATWALK gait analysis to study the abnormal gait characteristics of BCP rats by observing the maximum contact area, maximum contact maximum intensity, and mean intensity of the ipsilateral (left) limbs during free walking through a walkway. CATWALK gait analysis was performed to objectively assess mechanical allodynia in a chronic pain model, which has been reported in studies of inflammatory and neuropathic pain models [47, 48]. In our study, BCP rats showed a decrease in maximum contact area, maximum contact max intensity, and mean intensity of the ipsilateral (left) limb, thereby showing a decrease in limb availability, which was consistent with the results of Hu et al. [41], where they reported clinical findings of diminished function in daily life in patients with BCP.

Rosiglitazone is an agonist of PPAR-*γ*, which has insulin-sensitizing effects in diabetes and neuroprotective effects in neurological disorders. Previous *in vitro* studies have shown that rosiglitazone can reduce cell proliferation and induce apoptosis in various tumor phenotypes, including breast cancer [49, 50]. In our *in vitro* study, rosiglitazone inhibited the proliferation of Walker 256 breast cancer cells (used in our BCP model). To avoid the interference of tumor inhibition on our experimental results, intrathecal administration was conducted to ensure that the drug works only in the spinal cord and does not reduce Walker 256 cancer cell growth in the peripheral bone marrow. Previous studies have shown that intraperitoneal administration of rosiglitazone in a paclitaxel-induced pain model reduced nociceptive hyperalgesia in a dose-dependent manner [51]. In the present study, repeated intrathecal administration of rosiglitazone after successful establishment of the BCP model reversed mechanical allodynia in rats in a dose-dependent manner, suggesting an antinociceptive effect of rosiglitazone in the BCP model and providing direct evidence for the effect of rosiglitazone on the spinal cord. To further investigate the possible analgesic mechanism of rosiglitazone, we also intrathecally injected the PPAR-*γ* antagonist GW9662 to rats. Von-Frey and CATWALK gait analysis results showed that GW9662 reversed the analgesic effect of rosiglitazone in BCP rats. Subsequent molecular experiments also confirmed that intrathecal administration of rosiglitazone can significantly promote PPAR-*γ* protein expression in the spinal cord of BCP rats, whereas GW9662 reversed this effect. These results show that rosiglitazone exerts an antihyperalgesia effect by activating PPAR-*γ*.

Next, we explored the changes in endogenous expression and cellular localization of PPAR-*γ* in the spinal cord of BCP rats after cancer cell inoculation. We found that cancer cell inoculation significantly induced the expression of PPAR-*γ* in the spinal cord of BCP rats, which was consistent with the results of Gu et al. [52]. However, in the chronic constriction injury model, the total protein expression of PPAR-*γ* was unchanged in the spinal cord, whereas the expression of phosphorylated PPAR-*γ* was significantly downregulated [53]. In the paclitaxel-induced neuropathic pain model, PPAR-*γ* expression was significantly reduced in the spinal cord [51]. We speculated that the changes in the expression of PPAR-*γ* in the spinal cord may be caused by different models, which needs to be further studied. Moreover, the results of our immunofluorescence study showed that PPAR-*γ* colocalized primarily with neurons in the dorsal horn of the spinal cord, indicating that rosiglitazone relieves BCP by activating PPAR-*γ* in spinal cord neurons.

BCP is a type of complex pain that involves inflammatory pain and neuropathic pain [54]. NF-*κ*B and the NLRP3 inflammasomes are key components of inflammation. However, there has been controversy regarding the relationship between NF-*κ*B and NLRP3 inflammasome activation. In the present study, we found that the NF-*κ*B inhibitor, PDTC, attenuated nociceptive hyperalgesia and reversed BCP-induced p-NF-*κ*B and NLRP3 activation in BCP rats. Subsequent intrathecal injection of the NLRP3 inhibitor, MCC950, also showed the same analgesic effect. This suggests that cancer cell inoculation induced the activation of the NF-*κ*B/NLRP3 inflammatory axis in the spinal cord of BCP rats, which may be a potential target for the treatment of BCP. Similar to our findings, Fann et al. found that NF-*κ*B could promote the activation of the NLRP3 inflammasome in neurons after ischemic stroke [55]. However, in a spinal cord injury model [26], NF-*κ*B was not involved in NLRP3 activation in the spinal cord. We speculate that the different responses of NLRP3 inflammation to NF-*κ*B are highly dependent on the tissue microenvironments and cell types, which needs to be further investigated. Furthermore, Song et al. found that astragaloside IV ameliorates neuroinflammation-induced depression-like behaviors in mice via the PPAR-*γ*/NF-*κ*B/NLRP3 inflammasome axis. To explore whether there is a similar mechanism in the treatment of BCP with rosiglitazone, we performed drug interventions to observe these molecular changes. In this study, the expression of NF-*κ*B and NLRP3 was inhibited in the spinal cord of BCP rats after rosiglitazone treatment, which was reversed through treatment with the PPAR-*γ* inhibitor, GW9662. Moreover, the results of our immunofluorescence experiments showed that p-NF-*κ*B and NLRP3 colocalized primarily with neurons in the dorsal horn of the spinal cord. The results illustrate that rosiglitazone inhibits the NF-*κ*B/NLRP3 inflammatory axis through the activation of PPAR-*γ* in spinal cord neurons.

## 5. Conclusion

Collectively, our results demonstrate that rosiglitazone attenuates BCP through the activation of PPAR-*γ* to inhibit the NF-*κ*B/NLRP3 inflammatory axis in spinal cord neurons, which may be a promising therapeutic strategy for the treatment of BCP.

## Data Availability

The data that support the findings of this study are available from the corresponding author upon reasonable request.
